# *trans*-Cyclo­hex-2-ene-1,4-diyl bis­(4-nitro­phen­yl) dicarbonate

**DOI:** 10.1107/S1600536807065993

**Published:** 2007-12-18

**Authors:** Syed Nawazish Ali, Sabira Begum, Mitchell A. Winnik, Alan J. Lough

**Affiliations:** a H. E. J. Research Institute of Chemistry, International Center for Chemical Sciences, University of Karachi, Karachi 75270, Pakistan; b Department of Chemistry, University of Toronto, Toronto, Ontario, M5S 3H6, Canada

## Abstract

Although the title mol­ecule, C_20_H_16_N_2_O_10_, does not possess mol­ecular inversion symmetry, it lies on a crystallographic inversion centre which imposes disorder on the central cyclo­hexene ring. In addition, the cyclo­hexene ring has non-symmetry-related disorder over two sites, with the ratio of the major and minor components being 0.54:0.46. The overall effect is to produce four disorder components for the atoms of the cyclo­hexene ring. The side chain is perfectly ordered and the dihedral angle between the atoms of the carbonate group (O=CO_2_—) and the benzene ring is 72.99 (6)°.

## Related literature

For related literature, see: Ali *et al.* (2008 ▶); Ericsson & Hult (1991 ▶); Fréchet *et al.* (1986 ▶, 1987 ▶).
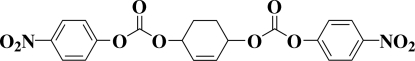


## Experimental

### 

#### Crystal data

C_20_H_16_N_2_O_10_
*M* *_r_* = 444.35
Monoclinic, 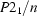

*a* = 5.6874 (4) Å
*b* = 13.4958 (10) Å
*c* = 12.7017 (5) Å
β = 96.453 (4)°
*V* = 968.76 (11) Å^3^
*Z* = 2
Mo *K*α radiation
μ = 0.13 mm^−1^
*T* = 150 (1) K
0.40 × 0.18 × 0.12 mm

#### Data collection

Nonius KappaCCD diffractometer
Absorption correction: multi-scan (*SORTAV*; Blessing, 1995 ▶) *T*_min_ = 0.560, *T*_max_ = 0.987
9286 measured reflections
2222 independent reflections
1408 reflections with *I* > 2σ(*I*)
*R*_int_ = 0.068

#### Refinement

*R*[*F* ^2^ > 2σ(*F* ^2^)] = 0.055
*wR*(*F* ^2^) = 0.167
*S* = 1.05
2222 reflections
185 parameters
58 restraints
H-atom parameters constrained
Δρ_max_ = 0.24 e Å^−3^
Δρ_min_ = −0.35 e Å^−3^

### 

Data collection: *COLLECT* (Nonius, 2002 ▶); cell refinement: *DENZO–SMN* (Otwinowski & Minor, 1997 ▶); data reduction: *DENZO–SMN*; program(s) used to solve structure: *SIR92* (Altomare *et al.*, 1994 ▶); program(s) used to refine structure: *SHELXTL/PC* (Sheldrick, 2001 ▶); molecular graphics: *PLATON* (Spek, 2003 ▶) ; software used to prepare material for publication: *SHELXTL/PC*.

## Supplementary Material

Crystal structure: contains datablocks global, I. DOI: 10.1107/S1600536807065993/hb2674sup1.cif

Structure factors: contains datablocks I. DOI: 10.1107/S1600536807065993/hb2674Isup2.hkl

Additional supplementary materials:  crystallographic information; 3D view; checkCIF report

## Figures and Tables

**Fig. 1.**
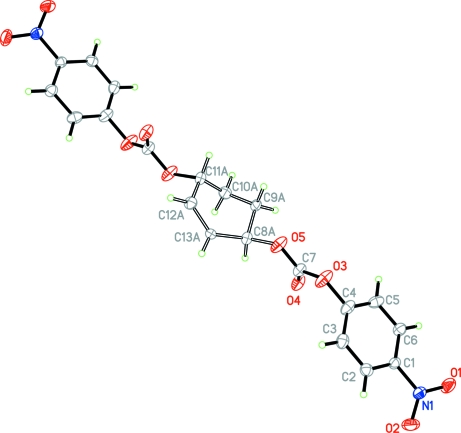
The molecular structure of (I) showing one component of disorder in the cyclohexene ring. Displacement ellipsoids drawn at the 30% probability level. Unlabeled atoms are related by the symmetry operator (1 - x, 1 - y, 1 - z).

**Fig. 2.**
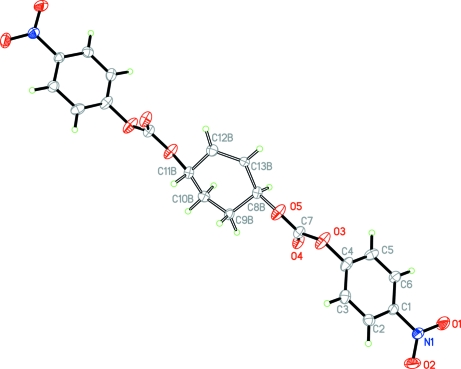
The molecular structure of (I) showing another component of disorder in the cyclohexene ring. Displacement ellipsoids drawn at the 30% probability level. Unlabeled atoms are related by the symmetry operator (1 - x, 1 - y, 1 - z).

## supplementary crystallographic information

### Comment

The title compound, (I), was synthesized two decades ago (Fréchet *et al.*, 1986) as a mixture of *cis* and *trans* isomers starting with a *cis* and *trans* mixture of cyclohex-2-ene-1,4-diol to obtain electrophilic character of diols. This compound has been used to obtain a wide variety of thermally and acid labile polymers for a variety of applications (Fréchet *et al.*, 1987; Ericsson & Hult, 1991). We have used the *trans* isomer of this alcohol for the synthesis of a number of homo and copolycarbonates (Ali *et al.*, 2008).

We report here the crystal structure of (I). Figures 1 and 2 show the two non-symmetry related components of disorder for the cyclohexene ring in (I). The crystallograhic inversion related disorder is not shown.

### Experimental

A solution of 4-nitrophenylchloroformate (1.41 g, 7.0 mmol) in dry dichloromethane (20 ml) was added dropwise *via* a 100 ml separating funnel into a solution of cyclohex-2-ene-1,4-diol (*trans* isomer) (0.40 g, 3.5 mmol) in anhydrous pyridine (0.49 g, 0.5 ml, 6.2 mmol) and dry dichloromethane (10 ml) in a 100 ml round-bottom flask. A white suspension appeared which was allowed to stir gently at room temperature for 12 h. After this time more dry dichloromethane (25 ml) was added, which dissolved the suspension and then the reaction mixture was stirred for another 6 h. Then it was quenched by adding deionized water (30 ml). The reaction mixture was transferred to a separating funnel (250 ml), and the lower organic phase was removed. The aqueous phase was washed with dichloromethane (20 ml × 2), and all the dichloromethane solutions were combined. These were then washed with deionized water (20 ml × 2), a 1.0% solution of acetic acid (30 ml × 2) and once more with deionized water (25 ml × 2), and then dried over anhydrous magnesium sulfate and filtered. After filtration, the solvent was removed by rotary evaporation. The product was dried in air overnight in a fume hood and then in a vacuum oven for 24 h at room temperature (< 1 Torr). The desired product was obtained in good yield (1.35 g, 86.5%) as a white crystalline solid. The product was recrystallized in dichloromethane and colourless needles of (I) were obtained by slow evaporation of solvent at room temperature. In addition to the X-ray structure determination, the structure of the crystalline sample was confirmed by Mass and NMR (^1^H and ^13^C)Spectroscopy.

### Refinement

All the hydrogen atoms were placed in calculated positions with C—H = 0.95 - 1.00 Å and refined as riding with *U*_iso_(H) = 1.2*U*_eq_(C). The components of the two symmetry independent disorder sites refined to 0.2680 (13) and 0.2320 (13). The disorder was modelled by creating two full rings for each component and by using suitable constraints and restraints to give each ring component similar geometries.

### Crystal data

**Table:**

C_20_H_16_N_2_O_10_	*F*_000_ = 460
*M**_r_* = 444.35	*D*_x_ = 1.523 Mg m^−^^3^
Monoclinic, *P*2_1_/*n*	Mo *K*α radiation λ = 0.71073 Å
Hall symbol: -P 2yn	Cell parameters from 9286 reflections
*a* = 5.6874 (4) Å	θ = 3–27.5º
*b* = 13.4958 (10) Å	µ = 0.13 mm^−^^1^
*c* = 12.7017 (5) Å	*T* = 150 (1) K
β = 96.453 (4)º	Needle, colourless
*V* = 968.76 (11) Å^3^	0.40 × 0.18 × 0.12 mm
*Z* = 2	

### Data collection

**Table:**

Nonius KappaCCD diffractometer	2222 independent reflections
Radiation source: fine-focus sealed tube	1408 reflections with *I* > 2σ(*I*)
Monochromator: graphite	*R*_int_ = 0.068
Detector resolution: 9 pixels mm^-1^	θ_max_ = 27.5º
*T* = 150(2) K	θ_min_ = 3.0º
φ scans and ω scans with κ offsets	*h* = −7→7
Absorption correction: multi-scan(SORTAV; Blessing, 1995)	*k* = −17→17
*T*_min_ = 0.560, *T*_max_ = 0.987	*l* = −16→16
9286 measured reflections	

### Refinement

**Table:**

Refinement on *F*^2^	Secondary atom site location: difference Fourier map
Least-squares matrix: full	Hydrogen site location: inferred from neighbouring sites
*R*[*F*^2^ > 2σ(*F*^2^)] = 0.055	H-atom parameters constrained
*wR*(*F*^2^) = 0.167	*w* = 1/[σ^2^(*F*_o_^2^) + (0.0883*P*)^2^ + 0.1379*P*] where *P* = (*F*_o_^2^ + 2*F*_c_^2^)/3
*S* = 1.05	(Δ/σ)_max_ = 0.001
2222 reflections	Δρ_max_ = 0.24 e Å^−^^3^
185 parameters	Δρ_min_ = −0.35 e Å^−^^3^
58 restraints	Extinction correction: none
Primary atom site location: structure-invariant direct methods	

### Special details

**Table:**

Geometry. All e.s.d.'s (except the e.s.d. in the dihedral angle between two l.s. planes) are estimated using the full covariance matrix. The cell e.s.d.'s are taken into account individually in the estimation of e.s.d.'s in distances, angles and torsion angles; correlations between e.s.d.'s in cell parameters are only used when they are defined by crystal symmetry. An approximate (isotropic) treatment of cell e.s.d.'s is used for estimating e.s.d.'s involving l.s. planes.
Refinement. Refinement of *F*^2^ against ALL reflections. The weighted *R*-factor *wR* and goodness of fit *S* are based on *F*^2^, conventional *R*-factors *R* are based on *F*, with *F* set to zero for negative *F*^2^. The threshold expression of *F*^2^ > σ(*F*^2^) is used only for calculating *R*-factors(gt) *etc*. and is not relevant to the choice of reflections for refinement. *R*-factors based on *F*^2^ are statistically about twice as large as those based on *F*, and *R*- factors based on ALL data will be even larger.

### Fractional atomic coordinates and isotropic or equivalent isotropic displacement parameters (Å^2^)

**Table:**

	*x*	*y*	*z*	*U*_iso_*/*U*_eq_	Occ. (<1)
N1	0.1105 (3)	0.66892 (13)	1.23307 (12)	0.0413 (4)	
O1	0.2311 (3)	0.72664 (14)	1.28816 (11)	0.0724 (5)	
O2	−0.0346 (3)	0.61480 (13)	1.26762 (11)	0.0677 (5)	
C1	0.1364 (3)	0.66467 (13)	1.12001 (13)	0.0337 (4)	
C2	−0.0422 (3)	0.62192 (14)	1.05264 (14)	0.0382 (4)	
H2	−0.1786	0.5955	1.0793	0.046*	
C3	−0.0189 (4)	0.61836 (14)	0.94586 (14)	0.0422 (5)	
H3	−0.1399	0.5899	0.8976	0.051*	
C4	0.1818 (4)	0.65644 (15)	0.91015 (14)	0.0431 (5)	
C5	0.3600 (3)	0.69951 (16)	0.97755 (15)	0.0455 (5)	
H5	0.4966	0.7254	0.9506	0.055*	
C6	0.3380 (3)	0.70468 (15)	1.08455 (14)	0.0406 (5)	
H6	0.4573	0.7347	1.1325	0.049*	
O3	0.1941 (3)	0.65488 (13)	0.80041 (10)	0.0582 (4)	
C7	0.3396 (3)	0.58846 (15)	0.76418 (14)	0.0394 (5)	
O4	0.4698 (2)	0.53485 (11)	0.81690 (10)	0.0491 (4)	
O5	0.3066 (3)	0.59665 (13)	0.65942 (10)	0.0551 (4)	
C8A	0.4218 (17)	0.5118 (9)	0.6065 (8)	0.0423 (9)	0.2680 (13)
H8A	0.4651	0.4583	0.6597	0.051*	0.2680 (13)
C9A	0.6450 (17)	0.5568 (7)	0.5726 (7)	0.0341 (18)	0.2680 (13)
H9A	0.7567	0.5721	0.6360	0.041*	0.2680 (13)
H9B	0.6059	0.6195	0.5341	0.041*	0.2680 (13)
C10A	0.7618 (16)	0.4853 (7)	0.5010 (6)	0.0509 (19)	0.2680 (13)
H10A	0.9225	0.5081	0.4897	0.061*	0.2680 (13)
H10B	0.7721	0.4177	0.5314	0.061*	0.2680 (13)
C11A	0.5965 (18)	0.4882 (9)	0.3987 (9)	0.0423 (9)	0.2680 (13)
H11A	0.6139	0.5519	0.3603	0.051*	0.2680 (13)
C12A	0.3392 (18)	0.4672 (7)	0.4093 (7)	0.037 (2)	0.2680 (13)
H12A	0.2298	0.4543	0.3487	0.045*	0.2680 (13)
C13A	0.2677 (17)	0.4674 (6)	0.5102 (5)	0.0435 (17)	0.2680 (13)
H13A	0.1188	0.4392	0.5203	0.052*	0.2680 (13)
C8B	0.496 (2)	0.5532 (8)	0.6018 (9)	0.0423 (9)	0.2320 (13)
H8B	0.6551	0.5605	0.6438	0.051*	0.2320 (13)
C9B	0.4298 (19)	0.4460 (8)	0.5879 (8)	0.0341 (18)	0.2320 (13)
H9C	0.2613	0.4410	0.5587	0.041*	0.2320 (13)
H9D	0.4489	0.4127	0.6578	0.041*	0.2320 (13)
C10B	0.5814 (19)	0.3938 (10)	0.5141 (8)	0.0509 (19)	0.2320 (13)
H10C	0.5527	0.3214	0.5139	0.061*	0.2320 (13)
H10D	0.7515	0.4062	0.5359	0.061*	0.2320 (13)
C11B	0.506 (2)	0.4379 (8)	0.4055 (9)	0.0423 (9)	0.2320 (13)
H11B	0.3474	0.4124	0.3759	0.051*	0.2320 (13)
C12B	0.5147 (19)	0.5479 (9)	0.3997 (9)	0.037 (2)	0.2320 (13)
H12B	0.5330	0.5814	0.3353	0.045*	0.2320 (13)
C13B	0.4943 (19)	0.5999 (9)	0.4934 (7)	0.0435 (17)	0.2320 (13)
H13B	0.4777	0.6698	0.4887	0.052*	0.2320 (13)

### Atomic displacement parameters (Å^2^)

**Table:**

	*U*^11^	*U*^22^	*U*^33^	*U*^12^	*U*^13^	*U*^23^
N1	0.0430 (9)	0.0476 (10)	0.0351 (8)	−0.0026 (8)	0.0119 (7)	−0.0027 (7)
O1	0.0743 (11)	0.1030 (14)	0.0427 (8)	−0.0408 (10)	0.0191 (7)	−0.0301 (8)
O2	0.0728 (11)	0.0881 (13)	0.0447 (9)	−0.0339 (10)	0.0181 (7)	0.0035 (8)
C1	0.0384 (10)	0.0327 (9)	0.0311 (9)	0.0052 (8)	0.0090 (7)	−0.0006 (7)
C2	0.0388 (10)	0.0342 (10)	0.0420 (10)	0.0021 (8)	0.0060 (8)	0.0018 (8)
C3	0.0487 (11)	0.0407 (11)	0.0357 (10)	0.0075 (9)	−0.0021 (8)	−0.0036 (8)
C4	0.0485 (11)	0.0510 (12)	0.0305 (9)	0.0232 (9)	0.0071 (8)	0.0028 (8)
C5	0.0383 (11)	0.0579 (13)	0.0431 (10)	0.0087 (9)	0.0164 (8)	0.0066 (9)
C6	0.0382 (10)	0.0470 (11)	0.0375 (10)	0.0010 (8)	0.0080 (7)	−0.0016 (8)
O3	0.0684 (10)	0.0769 (11)	0.0302 (7)	0.0395 (8)	0.0100 (6)	0.0045 (6)
C7	0.0377 (10)	0.0498 (11)	0.0306 (9)	0.0040 (9)	0.0033 (7)	−0.0026 (8)
O4	0.0554 (9)	0.0584 (9)	0.0326 (7)	0.0197 (7)	0.0006 (6)	−0.0061 (6)
O5	0.0578 (9)	0.0801 (11)	0.0278 (7)	0.0220 (8)	0.0059 (6)	−0.0007 (6)
C8A	0.052 (3)	0.043 (3)	0.0314 (14)	−0.0076 (18)	0.0046 (15)	−0.0078 (17)
C9A	0.040 (4)	0.037 (4)	0.025 (3)	−0.001 (3)	−0.001 (3)	−0.001 (2)
C10A	0.051 (4)	0.056 (4)	0.047 (4)	−0.019 (4)	0.013 (3)	−0.006 (3)
C11A	0.052 (3)	0.043 (3)	0.0314 (14)	−0.0076 (18)	0.0046 (15)	−0.0078 (17)
C12A	0.037 (4)	0.041 (4)	0.032 (4)	0.002 (3)	−0.002 (3)	0.004 (3)
C13A	0.061 (4)	0.039 (3)	0.032 (3)	−0.024 (3)	0.013 (3)	−0.004 (2)
C8B	0.052 (3)	0.043 (3)	0.0314 (14)	−0.0076 (18)	0.0046 (15)	−0.0078 (17)
C9B	0.040 (4)	0.037 (4)	0.025 (3)	−0.001 (3)	−0.001 (3)	−0.001 (2)
C10B	0.051 (4)	0.056 (4)	0.047 (4)	−0.019 (4)	0.013 (3)	−0.006 (3)
C11B	0.052 (3)	0.043 (3)	0.0314 (14)	−0.0076 (18)	0.0046 (15)	−0.0078 (17)
C12B	0.037 (4)	0.041 (4)	0.032 (4)	0.002 (3)	−0.002 (3)	0.004 (3)
C13B	0.061 (4)	0.039 (3)	0.032 (3)	−0.024 (3)	0.013 (3)	−0.004 (2)

### Geometric parameters (Å, °)

**Table:**

N1—O1	1.208 (2)	C9A—H9A	0.9900
N1—O2	1.220 (2)	C10A—C11A	1.516 (10)
N1—C1	1.461 (2)	C10A—H10A	0.9900
C1—C2	1.379 (3)	C10A—H10B	0.9900
C1—C6	1.387 (3)	C11A—O5^i^	1.500 (10)
C2—C3	1.378 (3)	C11A—C12A	1.511 (10)
C2—H2	0.9500	C11A—H11A	1.0000
C3—C4	1.374 (3)	C12A—C13A	1.387 (11)
C3—H3	0.9500	C12A—H12A	0.9500
C4—C5	1.379 (3)	C13A—H13A	0.9500
C4—O3	1.403 (2)	C8B—C9B	1.502 (11)
C5—C6	1.381 (3)	C8B—C13B	1.513 (10)
C5—H5	0.9500	C8B—H8B	1.0000
C6—H6	0.9500	C9B—C10B	1.517 (11)
O3—C7	1.336 (2)	C9B—H9C	0.9900
C7—O4	1.187 (2)	C9B—H9D	0.9900
C7—O5	1.327 (2)	C10B—C11B	1.519 (11)
O5—C8B	1.491 (10)	C10B—H10C	0.9900
O5—C11B^i^	1.491 (10)	C10B—H10D	0.9900
O5—C11A^i^	1.500 (10)	C11B—C12B	1.489 (11)
O5—C8A	1.515 (9)	C11B—O5^i^	1.491 (10)
C8A—C9A	1.513 (10)	C11B—H11B	1.0000
C8A—C13A	1.543 (9)	C12B—C13B	1.397 (13)
C8A—H8A	1.0000	C12B—H12B	0.9500
C9A—C10A	1.527 (10)	C13B—H13B	0.9500
			
O1—N1—O2	122.74 (16)	O5^i^—C11A—C12A	108.3 (7)
O1—N1—C1	118.76 (16)	O5^i^—C11A—C10A	100.1 (7)
O2—N1—C1	118.49 (16)	C12A—C11A—C10A	115.6 (9)
C2—C1—C6	122.64 (17)	O5^i^—C11A—H11A	110.8
C2—C1—N1	118.54 (16)	C12A—C11A—H11A	110.8
C6—C1—N1	118.82 (16)	C10A—C11A—H11A	110.8
C3—C2—C1	118.65 (18)	C13A—C12A—C11A	118.0 (9)
C3—C2—H2	120.7	C13A—C12A—H12A	121.0
C1—C2—H2	120.7	C11A—C12A—H12A	121.0
C4—C3—C2	119.16 (18)	C12A—C13A—C8A	122.2 (9)
C4—C3—H3	120.4	C12A—C13A—H13A	118.9
C2—C3—H3	120.4	C8A—C13A—H13A	118.9
C3—C4—C5	122.15 (17)	O5—C8B—C9B	104.4 (9)
C3—C4—O3	117.26 (18)	O5—C8B—C13B	110.5 (8)
C5—C4—O3	120.50 (19)	C9B—C8B—C13B	108.5 (10)
C4—C5—C6	119.38 (18)	O5—C8B—H8B	111.0
C4—C5—H5	120.3	C9B—C8B—H8B	111.0
C6—C5—H5	120.3	C13B—C8B—H8B	111.0
C5—C6—C1	118.01 (18)	C8B—C9B—C10B	111.5 (9)
C5—C6—H6	121.0	C8B—C9B—H9C	109.3
C1—C6—H6	121.0	C10B—C9B—H9C	109.3
C7—O3—C4	116.99 (14)	C8B—C9B—H9D	109.3
O4—C7—O5	128.71 (18)	C10B—C9B—H9D	109.3
O4—C7—O3	125.86 (17)	H9C—C9B—H9D	108.0
O5—C7—O3	105.43 (16)	C9B—C10B—C11B	105.0 (9)
C7—O5—C8B	115.5 (5)	C9B—C10B—H10C	110.7
C7—O5—C8A	111.3 (5)	C11B—C10B—H10C	110.7
C9A—C8A—O5	104.0 (8)	C9B—C10B—H10D	110.7
C9A—C8A—C13A	110.5 (8)	C11B—C10B—H10D	110.7
O5—C8A—C13A	114.2 (7)	H10C—C10B—H10D	108.8
C9A—C8A—H8A	109.3	C12B—C11B—O5^i^	104.8 (8)
O5—C8A—H8A	109.3	C12B—C11B—C10B	115.4 (11)
C13A—C8A—H8A	109.3	O5^i^—C11B—C10B	103.6 (8)
C8A—C9A—C10A	110.5 (8)	C12B—C11B—H11B	110.9
C8A—C9A—H9A	109.5	O5^i^—C11B—H11B	110.9
C10A—C9A—H9A	109.5	C10B—C11B—H11B	110.9
C11A—C10A—C9A	103.0 (8)	C13B—C12B—C11B	116.9 (11)
C11A—C10A—H10A	111.2	C13B—C12B—H12B	121.6
C9A—C10A—H10A	111.2	C11B—C12B—H12B	121.6
C11A—C10A—H10B	111.2	C12B—C13B—C8B	125.0 (11)
C9A—C10A—H10B	111.2	C12B—C13B—H13B	117.5
H10A—C10A—H10B	109.1	C8B—C13B—H13B	117.5

Symmetry codes: (i) −*x*+1, −*y*+1, −*z*+1.

## References

[bb1] Ali, S. N., Ghafouri, S., Yin, Z., Froimowicz, P., Begum, S. & Winnik, M. A. (2008). In preparation.

[bb2] Altomare, A., Cascarano, G., Giacovazzo, C., Guagliardi, A., Burla, M. C., Polidori, G. & Camalli, M. (1994). *J. Appl. Cryst.* **27**, 435.

[bb3] Blessing, R. H. (1995). *Acta Cryst.* A**51**, 33–38. 10.1107/s0108767394005726 7702794

[bb4] Ericsson, J. & Hult, A. (1991). *Makromol. Chem.* **192**, 1609–1619.

[bb5] Fréchet, J. M. J., Bouchard, F., Eichler, E., Houlihan, F. M., Ilzawa, T., Kryczka, B. & Willson, C. G. (1987). *Polym. J.* **19**, 31–49.

[bb6] Fréchet, J. M. J., Bouchard, F., Houlihan, F. M., Eichler, E., Ktyczka, B. & Willson, C. G. (1986). *Macromol. Chem. Rapid Commun.* **7**, 121–126.

[bb7] Nonius (2002). *COLLECT* Nonius BV, Delft, The Netherlands.

[bb8] Otwinowski, Z. & Minor, W. (1997). *Methods in Enzymology*, Vol. 276, *Macromolecular Crystallography*, Part A, edited by C. W. Carter Jr & R. M. Sweet, pp. 307–326. New York: Academic Press.

[bb9] Sheldrick, G. M. (2001). *SHELXTL/PC* Version 6.1. Bruker AXS Inc., Madison, Wisconsin, USA.

[bb10] Spek, A. L. (2003). *J. Appl. Cryst.* **36**, 7–13.

